# From Autologous Bone Tissue to Bioengineered Material Solutions in Post-Traumatic Orbital Wall Reconstruction: An Overview

**DOI:** 10.3390/jfb16120430

**Published:** 2025-11-24

**Authors:** Ovidiu Lazăr, Gerhard Garhoefer, Diana Ionescu, Tudor Ionescu, Sînziana Istrate, Alina Popa-Cherecheanu, Dana Galieta Mincă

**Affiliations:** 1 Carol Davila University of Medicine and Pharmacy, 020021 Bucharest, Romania; ovidiu.lazar@drd.umfcd.ro (O.L.); dana.minca@umfcd.ro (D.G.M.); 2 Department of Anesthesia and Critical Care, Life Memorial Hospital, 010719 Bucharest, Romania; 3 Department of Clinical Pharmacology, Medical University of Vienna, 1090 Vienna, Austria; gerhard.garhoefer@meduniwien.ac.at; 4 Titu Maiorescu University, 040051 Bucharest, Romania; diana.ionescu@prof.utm.ro (D.I.); tudor.ionescu@prof.utm.ro (T.I.); 5 Victor Gomoiu Children Clinical Hospital, 022102 Bucharest, Romania; 6 Bine Clinic, 020483 Bucharest, Romania; 7 Department of Ophthalmology, Emergency University Hospital, 050013 Bucharest, Romania

**Keywords:** orbital wall reconstruction, bioengineered solutions, technological innovations

## Abstract

Orbital wall fractures are a common consequence of trauma-related craniofacial injuries. Multistage treatment and poor functional and aesthetic results render the reconstruction of an orbit extremely challenging. Advances in surgical technologies, imaging software, and biomaterials have continuously improved outcomes. The choice of materials plays a critical role in patient outcomes. Over time, the type of material involved advanced from autografts (autologous tissues such as bone grafts and muscle flaps) to allografts (metals, ceramics, plastic materials, or combinations of these materials). In this study, we provide a comprehensive overview of the latest scientific insights, including the advantages and disadvantages of each material used in terms of stability, cost, safety, biocompatibility, durability, and intraoperative readiness. Bioengineered solutions seem to be the future of orbital wall reconstruction; both material and technological innovations hold promise for further advancements.

## 1. Introduction

Traumatic injuries of the craniofacial area have become an increasingly significant medical issue over the past two decades [1]. There is a wide range of etiologies, but currently, a bimodal distribution of frequencies among age groups persists: domestic-related and motorcycle accidents in adults and sport-related causes in teenagers and children [2,3]. To achieve long-term full functionality and a low rate of complications, current proper management imposes clinical, computer-AI-assisted imagistic evaluations; optimal timing for delicate and complex surgical procedures to restore the function and aesthetic appearance of the orbital area and the eye; and the correct choice of material for reconstructions [4]. The ideal biomaterial [5] for reconstruction with respect to orbital fractures must possess certain characteristics: biocompatibility, long-term stability, mechanical endurance, safety, low cost, and intraoperative readiness. Recent studies underline the increasing interest in the use of alloplastic materials, especially for new bioengineered solutions that can produce customized prosthetic devices in close proximity to the operating room.

A comprehensive literature search was conducted in PubMed, Scopus, and Web of Science databases for publications between January 2000 and October 2025, using combinations of the terms “orbital wall reconstruction”, “biomaterials”, “3D printing”, and “bioengineered scaffolds”. Inclusion criteria comprised peer-reviewed studies, clinical trials, systematic reviews, and meta-analyses focusing on post-traumatic orbital wall repair. Experimental animal models were included if they provided translational data. Exclusion criteria involved purely aesthetic orbital procedures, non-traumatic reconstructions, and non-English publications. Reference lists of relevant articles were manually screened to ensure completeness.

## 2. Types of Materials Used for Orbital Reconstruction

A multitude of osteosynthetic materials have been used for the reconstruction of the walls of the orbit after post-traumatic fractures (Table 1) [6].

The medical literature describes a shifting preference for certain materials, with each decade favoring a different one as “ideal”. In the current sense, the ideal characteristics of the materials used for orbital wall reconstruction must include the following: biocompatibility, long-term stability and mechanical resistance, low cost, immediate availability, minimal risk of late complications or need for reintervention, and overall safety. Currently, existing materials have advantages and disadvantages, and they have specific indications, but none can be considered ideal.

Bioengineered materials appear to be the next stage in orbital reconstruction materials [7].

### 2.1. Biological Materials

#### 2.1.1. Autologous Bone

The use of autologous bone tissue was the first choice of bone defect reconstruction material and remained the gold standard for restoring the walls of the orbit in the early period of the modern era [8]. The arguments in favor of its use are as follows: relative ease of procurement, low cost, and instant availability. Rare undesirable effects, such as complications at the donor site (i.e., infection, extrusion, collagenous capsule formation, ocular tethering) or the resorption of the grafted material, have reduced the use of this material [9]. Bone tissues can come from the sternum, mandible, tibia, scapula, iliac crest, and calvaria.

Split calvarial bone appears to be a suitable option for orbital wall reconstruction, as it is readily accessible, allows variable graft sizes, and results in a scar that can be easily concealed within hair-bearing areas [10]. In a study conducted on 9650 cases of craniofacial and maxillofacial reconstruction, Tessier et al., using various surgical techniques and parietal bone grafts, reported favorable results immediately and over time with respect to both in situ and ex vivo split techniques [11]. In a prospective study involving 24 patients with orbital wall fractures, using bone autografts from the iliac crest, Kontio et al. obtained very good results; they reported an increased rate of bone resorption, but this was accompanied by advantageous remodeling, and only one patient (4%) presented with enophthalmos and hypophthalmos [12]. In 1992, Girdler and Hosseini published the first case of orbital reconstruction using autologous bone grafts harvested from the lingual mandibular cortex. The authors concluded that this technique is easy to implement, the collection of autologous material occurs rapidly, and postoperative complications are reduced [13]. In a 75-case study conducted by Kosaka et al. in 2004, focusing on the efficacy of using bone grafts from the outer cortex of the mandible for reconstructing orbital walls in patients with blow-out fractures, the authors concluded that this technique has several advantages: ease of harvest, appropriate size and curvature, absence of functional disability, no visible scars, and rare major complications [14].

Exploring the techniques and materials available for orbito-zygomatic area reconstruction in a review published in January 2024, Reiss et al. emphasized the importance of using autologous bone materials; these materials are widely available, but they also exhibit disadvantages related to early resorption and potential donor site complications. Simultaneously, their findings critically highlight the inconsistent results arising from the use of alloplastic materials and open a window of opportunity to the new solutions offered by regenerative medicine and its related bioengineering techniques [15].

#### 2.1.2. Autologous Cartilage

Autologous cartilaginous tissue has been explored as an alternative to bone tissue for orbital floor reconstruction because it is easily available, malleable, and does not undergo the resorption process [16]. Cartilage grafts are more commonly harvested from the nasal septum, concha, or ear canal [17]. The collection of these grafts from the nasal septum can be carried out rapidly; the cosmetic effect is optimal, and local complications are minimized. The cartilaginous tissue has a low metabolic rate and rich vascularization, which allows increased viability and rapid regeneration, favoring the viability of the graft [17].

In a recent systematic review, Abukhder et al. investigated the use of autologous cartilaginous grafts in orbital fracture reconstructions carried out in 259 patients. Conchal, auricular, naso-septal, and costal grafts were harvested. Complications were observed in 13.9% of patients, most commonly including diplopia, infraorbital para/anesthesia, and enophthalmos. There was no graft failure or morbidity at the collection site. This type of material provides adequate structural support for the contents of the orbit, and it can be easily harvested, shaped, and positioned [18]. The cartilaginous graft is not radiopaque and does not provide mechanical support similar to bone tissues, and its use is limited to small structural defects [19].

#### 2.1.3. Allografts

Allografts are tissue grafts of human origin; however, they differ in that they are from individuals with different genotypes. Bone allografts are the most common substitute for autografts in bone growth procedures carried out in the USA [20]. Their use involves controlling the donor’s pathology as directly as possible, which is a procedure implemented by the tissue bank. The donor must undergo specific medical screening [21]. There are some chemical or physical methods for removing immune reactivity to eliminate the risk of triggering the host versus graft reaction, but none are infallible [22]. Over time, these biomaterials are reabsorbed and replaced by new bone [23]. Lyophilized dura mater, demineralized human bone, lyophilized cartilage, and irradiated fascia lata are among the most widely used allograft materials in the reconstruction of post-traumatic orbital defects. A retrospective study on the use of demineralized bone grafts for the reconstruction of orbit and craniofacial defects carried out by Neigel et al. revealed excellent results, without complications attributed to the use of this type of graft. The lack of autologous material harvesting sites reduces perioperative risks [24].

#### 2.1.4. Xenografts

Although several variants of non-human biological materials have been tested over time (collagen membrane, porcine sclera, porcine skin gelatin, and bovine bone), studies lacking statistical significance have produced inconsistent and non-reproducible results. In a 2021 study of 10 subjects with post-traumatic orbital wall fracture led by Huang et al., pig bone matrices decellularized by supercritical CO_2_ (ABCcolla^®^) were used. The results were safe and efficient, exhibited high stability and biocompatibility, and produced a low rate of infectious complications [25]. However, the conclusions of this study were based on only an 8-week post-procedural follow-up and did not address the possibility of transmissible diseases associated with the use of xenografts. The use of xenografts in orbit reconstruction is not encouraged due to the possible transmission of infectious diseases, immunological rejection, and the high and unpredictable risk of reabsorption [26].

In summary, biological grafts, including autologous, allogenic, and xenogeneic tissues, have historically provided biocompatible and accessible options for orbital wall reconstruction. However, they are limited by donor-site morbidity, potential immunogenicity, and variable resorption rates. These shortcomings have prompted a progressive shift toward synthetic alloplastic and composite substitutes that offer more predictable outcomes and eliminate donor-related risks.

### 2.2. Alloplastic Materials

Transitional metals (titanium and cobalt alloys) are the oldest solutions with the best long-term results for the reconstruction of craniofacial defects [27]. Recently, other ceramic alloplastic materials (bioactive ceramic glass, calcium phosphate, hydroxyapatite, tricalcium β phosphate, aluminum phosphate, and calcium sulphate), polymers, and composite materials have been widely used, with several specific advantages and disadvantages [28].

#### 2.2.1. Titanium

Titanium and most of its alloys (vanadium, aluminum, and niobium) exhibit good biocompatibility with respect to human tissues, and they are practically inert from a pathophysiological point of view [27]. They have been used in medical practice since the 1950s [29]. Their high resistance to corrosion, ability to form a biofilm on the surface, which induces the natural adsorption of proteins while maintaining their natural conformation, and optimal reactivity, which induces the formation of calcium phosphate, render titanium and its alloys the most biocompatible metal [30]. This advanced compatibility can be amplified via special techniques for processing prosthetic surfaces, such as plasma spraying, sandblasting, and laser treatment. These treatments increase the roughness of the surfaces and thus amplify cellular adhesion, resulting in the perfect osseointegration of implants into the bone and creating premises for reducing complications—such as infection, inflammation, and aseptic non-integration of the implant [31]. The toxicity of titanium is relatively low, but the presence of alloy components can induce serious side effects, such as aluminum-induced nephro- and neurotoxicity [32]. Physical characteristics such as machinability, mechanical strength, and elasticity, combined with biocompatibility, render titanium the most successful metallic material to ever be applied in the field of biomedical engineering [33]. Titanium alloys are found in different commercial forms (plates, screws, and meshes), with varying geometric appearances and standard dimensions [28]. The use of standard titanium prosthetic materials is not the best technical or economic solution. The high cost of titanium alloys and the difficulty of removing prostheses embedded in the bone tissue adjacent to the lesion in the case of reintervention (infection and dimensional incongruence due to the development of pediatric patients) necessitate the use of highly personalized prosthetic materials. Although the customized solutions obtained through computer-based assistance seem to be the state of the art, they require additional resources [34]. In a retrospective study of unilateral orbital reconstruction involving ninety subjects, Canzi et al. re-shaped standard meshes using an intraoperative protocol based on six anatomical landmarks. The results obtained were satisfactory. The incidence of complications and reinterventions was reduced, with the author recommending “it should be a skill of all surgeons who deal with orbital wall reconstruction in daily clinical activity” [35]. However, the intraoperative modeling of titanium mesh plates to achieve a perfect fit for the patient’s orbital defect is time-consuming and a significant source of error, especially in cases where surgeons have little experience. In addition, excessive modeling could result in a decrease in the prosthetic material’s mechanical strength, and there is a risk of clinical complications such as implant breakage, loosening of screws, and bone resorption [36,37,38].

Due to the geometric variability of craniozygomatic lesions and economic factors, modular commercial forms have replaced pre-formed implants [39]. However, over the past decade, individualized solutions have become increasingly available [40].

The cost of producing customized prostheses has decreased, and technological solutions have diversified and become available globally [41]. Direct metal laser sintering is an innovative technique that allows for the creation of customized titanium meshes [42]. The rapid availability of customized prostheses reduces the time and risks associated with the surgical act, especially in the case of geriatric patients, as increased age is associated with more complex orbital fractures, increased comorbidities (i.e., chronic anticoagulant or antiaggregant medication resulting in bleeding), and higher costs [43,44].

#### 2.2.2. Biological Ceramics

Hydroxyapatite became available for bone reconstructive surgery in the 1990s [45]. Its chemical structure is identical to that of the bone’s mineral substances. Some studies have attempted to test nanostructured hydroxyapatite as an augmenting material to expand the orbit following the initial placement of a porous implant [46]. However, other studies have shown its inferiority in orbital wall reconstructive surgery compared to polymer materials [47]. Recent research on the physical or chemical modification of nano-hydroxyapatite surfaces [48], in addition to the production of composite materials by combining it with natural or synthetic polymers, has created new reconstructive opportunities, the efficiency and safety of which still require investigation in further studies [49].

#### 2.2.3. Polymers

Although titanium-based implants are still used, their disadvantages—such as the need for surgical removal in complicated cases, the resulting translocation of bone growth in children, and radiological opacity—have resulted in alternative solutions: bioresorbable polymers [50,51]. Polymers are macromolecules comprising small repetitive units. These include natural polymers (silk, cellulose, chitosan, collagen, alginate, and fucoidan) or synthetic polymers (polyethylene and polyurethanes), and they can be resorbable or non-resorbable and porous or non-porous [52].

##### Non-Absorbable Permanent Polymer Implants

Porous ultra-high-density polyethylene (PE; Medpor) is an alloplastic material that is globally used in craniofacial reconstructions. Sheets of various sizes and thicknesses have been used to cover small defects since the 1990s [53]. This material can be easily shaped, and connective tissue and neoformation vessels can readily develop in the pores of this prosthetic material type. It is highly biocompatible, with rejection reactions being rare [54]. In a study conducted on 285 grafts used in 187 subjects—dedicated to the evaluation of complications and risk factors associated with the use of Medpore in craniofacial reconstruction—Cenzi et al. emphasized that unfavorable outcomes depend on the anatomical site of the intervention and the presence of previous interventions [55].

Although silicone is easy to handle, flexible, and relatively inexpensive, the unacceptably high rate of local complications in the immediate postoperative period has resulted in the sporadic use of this material [56,57]. In a recently published manuscript, Takabayasi et al. described the use of silicone in the reconstruction of the inferior–medial wall of the orbit, highlighting the need for early removal—within 1 month—of the synthetic material’s application to prevent postoperative complications [58].

##### Absorbable Polymer Implants

The use of resorbable polymer-based osteosynthesis materials dates back to the 1990s, although the technology for their production had been known three decades earlier. Although resorbable materials are biocompatible, their resorption causes host-versus-graft reactions that can result in fistulas, osteolysis, and persistent edema at the implant site [59]. Other disadvantages of this type of material include low mechanical strength and limited ability to be molded to fit the specific requirements of the defect [59]. These disadvantages offset the benefits of the material, such as its lack of radio-opacity, lower potential to induce osteopenia, and absence of corrosion issues associated with metals [60].

The resorbable polymers used in facial reconstructive surgery are mainly represented by polyacids: polyglycolic acid (PGA), poly-L-lactic acid (PLLA), poly-D-lactic acid (PDLA), and their copolymers [61]. Other chemical structures are also useful; one versatile example is polydioxanone (PDS), an ester–ether cyclic polymer [62]. In a retrospective study conducted on 189 subjects between 2003 and 2007, with the aim of investigating the indications, surgical techniques, and materials used for orbital reconstruction, Gosau et al. highlighted that PDS was used in 70.5% of cases, compared to titanium mesh in only 6.2% of cases. The rate of complications was 19%, including one case of complete blindness [63]. Almost two decades later, PDS was used in 41% of orbital wall reconstructions compared to 18% for titanium mesh, and 3D printing technology provided customized solutions in 75% of cases, as shown in a recent online survey led by Burger et al. [64]. Titanium mesh remains the most used reconstructive material in large orbital defects, while resorbable polymers (PLLA/PDLA and PDS) are used in the reconstruction of small-sized or medium-sized defects. Both alloplastic solutions exhibit lower enophthalmos-type or diplopia complications, as evidenced by a meta-analysis published in 2021 by Bourry et al. [65].

#### 2.2.4. Composite Materials

Composite materials are used in orbital wall reconstruction to improve mechanical and biological properties. The ideal material should combine the advantages of each component; offer temporal durability and perfect biocompatibility; eliminate unwanted complications; and be cost-effective and readily available. Recent bioengineering solutions have attempted to meet these requirements, but no material has yet proven to be ideal [66].

Overall, composite materials combine the strength of metals with the adaptability of polymers and ceramics, achieving superior mechanical stability and osteoconductivity. Nevertheless, their higher production costs and limited long-term clinical validation currently restrict their widespread adoption. Future bioengineered composites may overcome these barriers by integrating smart surface chemistry and controlled biodegradability [7].

Recent studies (Kim et al., 2025; Maher et al., 2022; Taxis et al., 2023) [67,68,69] consistently show no single material achieving superiority across all outcomes. Titanium meshes remain most stable for large defects, with lower rates of implant displacement and enophthalmos (<5%), whereas resorbable polymers exhibit fewer secondary surgeries but higher rates of transient diplopia (10–15%). Composite and hybrid materials achieve promising integration with lower infection rates (<2%) but lack long-term data. These comparative findings reinforce the current absence of an ‘ideal’ reconstructive material.

#### 2.2.5. Analytical Summary—Materials Versus Clinical Outcomes

**Figure 1:**
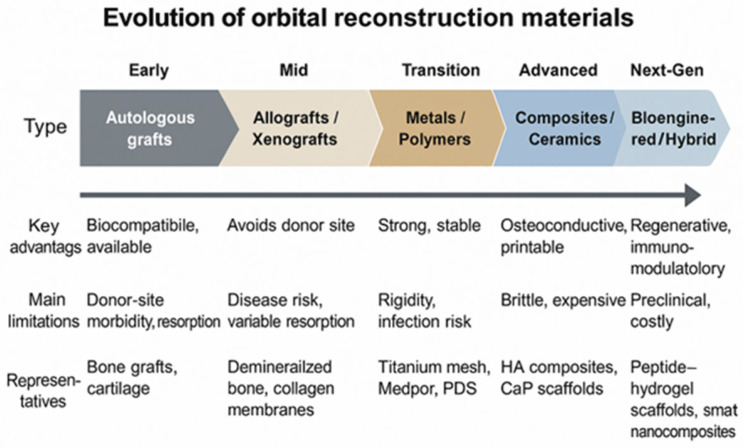
Evolution of orbital wall reconstruction materials.

The evolution of orbital wall reconstruction materials reflects a continuous balance between biological compatibility and mechanical reliability. Autologous tissues remain biocompatible and cost-effective but suffer from resorption and donor-site morbidity, limiting long-term volumetric stability. Allografts and xenografts reduce harvesting risks but exhibit inconsistent integration and potential immunogenicity. Metallic implants, especially titanium meshes, provide unmatched structural stability and radiographic visibility, though their rigidity and cost may require reconsideration in pediatric or thin-tissue contexts. Resorbable polymers offer the advantage of gradual degradation and reduced reoperation rates, but can provoke inflammatory responses during resorption. Composite and bioactive ceramic materials bridge the mechanical–biological divide, promoting osteoconduction and customization through 3D printing. Finally, hybrid and bioengineered systems integrate cellular and immunomodulatory components, aiming to couple early mechanical support with long-term biological regeneration. These trajectories underline a clear translational shift from passive mechanical repair toward active biological restoration of orbital wall integrity (Figure 1).

Conceptual schematic summarizing the transition from autologous grafts to next-generation bioengineered materials used in orbital wall reconstruction.

### 2.3. Patient-Specific Implants

Patient-specific implants (PSIs) are a new personalized medicine concept observed in all medical branches. New materials that are biocompatible, radio-opaque, and more stable than manually bent titanium are now available on the market [70]. The reconstruction of complex and extensive orbital fractures benefits from the development and implementation of digital technology—such as computer-assisted surgery—and the optimization of procedural treatment before, during, and after the surgical procedure [71]. The production of PSIs using computer-aided techniques that compare the affected area with the healthy contralateral one offers reconstructive solutions in a limited number of cases [72]. Recently, a systematic review summarized the latest literature on the use of extended reality—including augmented reality (AR), mixed reality (MR), and virtual reality (VR)—in preoperative planning for orbital fractures. These advanced imaging techniques favor obtaining a PSI that is better integrated into the reconstructed anatomical structures compared to techniques based on classical three-dimensional imaging [73].

In recent years, the production of titanium mesh PSIs via 3D printing (PSI 3DP) techniques has been increasingly used [74]. Many published studies highlight the advantages of PSI 3DP in terms of stability, ease of use, reduced surgery time, and improved functional outcomes [75]. In a systematic review and meta-analysis, Kumar Sing et al. compared pre-shaped implants on a PSI 3DP model (281 subjects) to manual free-hand shaping (MFS) (283 patients) in orbital wall reconstructions. Despite the overall high risk of bias, PSI 3DP models resulted in improved accuracy with respect to fit and defect area coverage, and they exhibit improved enophthalmos and diplopia correction. The 3DP models appear to be valuable for accurate orbital wall reconstructions, with fewer complications than those for conservative free-hand-shaped implants [76]. In another related study involving only low-bias-risk adults—which only comprised a systematic review and meta-analysis—Kotecha et al. stressed that although there is a tendency to consider PSIs as a better technical solution in terms of reducing operation times and decreasing postoperative complications such as enophthalmos, no statistically significant differences in key outcomes were identified. The study concluded that, based on the results obtained, the choice of the implant used should be left to the discretion of the surgeon [68]. However, as 3D-printers and their materials become more accessible and inexpensive, the existence of a “point of care” that offers PSIs for orbital reconstruction within the proximity of the operating room will become a necessity [77].

Patient-specific implants represent a major leap toward personalized orbital reconstruction, improving surgical precision and reducing operative time. Despite these advantages, their use is still limited by equipment costs, regulatory constraints, and the need for high-resolution imaging and technical expertise. As 3D-printing technologies become more affordable, PSIs will likely become the clinical standard for complex orbital defects.

## 3. Recent Discoveries

Recent data regarding the reconstruction of craniomaxillofacial bones indicate the emergence of new specialized sciences with medical applications, with bone tissue engineering (BTE) being one of them [78]. BTE is an overlapping science that combines bioengineering, cell transplantation, and materials science to provide biological alternatives for fracture repair. By creating an environment that mimics the natural biological one, stimulating the regeneration and proliferation of normal tissue and cells, BTE could represent a solution in fracture repair [79]. Cells, growth factors, and biological frames are the necessary components required for tissue engineering [80].

Clinical evidence that has emerged in the last decade highlights the promise of hybrid nanocomposite bioactive materials and the ongoing research activities toward the development of multifunctional or stimuli-responsive implants [81]. Bioactive peptide-hydrogel scaffolds induce osteogenesis and osteoimmunomodulation, promoting bone regeneration [82].

The interaction between macrophages and mesenchymal stem cells (MSCs) forms the biological basis of bone regeneration, involving collaborative mechanisms such as adhesion, migration, immunological modulation, and osteogenic activity [83]. Several in vitro and in vivo studies have been conducted to study the osteogenic effect of some biologically active peptides (Table 2) [84].

Modulating immune system cells to augment the osteogenic capabilities of MSCs represents a potential new bone repair method [85]. The development of reconstructive scaffolds (i.e., hydrogels, demineralized bone, bioresorbable polymers, bio ceramics, HA ceramics) loaded with biological factors—such as bone morphogenetic protein-2 (BMP-2), transforming growth beta factor (TGF-beta), vascular endothelial growth factor (VEGF), stromal cell-derived factor-1 (SDF-1), and fibroblast growth factor obtained by the symbiotic culture of macrophage cells and MSCs—is under investigation [83]. In a recent study, Niu et al. emphasized the ability of HA to promote the osteogenic transformation of MSCs by favoring the increased expression of osteo- and angiogenic genes secondary to the local release of calcium ions and phosphates [80]. This increased ability to induce the angiogenesis of HA is augmented by the inclusion of inorganic materials (e.g., copper, magnesium, iron, strontium, other dopant metals, concave and convex nanorods), natural polymers (collagen, chitosan, and gelatin), and synthetic polycaprolactone and polylactic acid in its structure [80].

Conventionally, almost all materials can be improved by modifying their outermost layer—physically or chemically—and new materials and geometries can be created with more intricate characteristics [86]. Physical modifications include grit-blasting, etching, and ultrasound blasting. Chemical procedures include atomic-layer deposition, ion infusion, oxidation, carbiding, and nitriding of the surface [87]. There is evidence that cells are responsive to their environment and can react to the shape of their surroundings [86]. Surface roughness and porosity additionally stimulate cell adhesion and osteosynthesis [88].

Recent discoveries in bone tissue engineering highlight the growing synergy between material science, cellular biology, and immunomodulation. Although most data remain preclinical, these approaches show great promise in achieving controlled osteogenesis and faster healing. Translating these laboratory findings into reproducible, safe, and cost-effective clinical protocols remains the next frontier in orbital reconstruction.

## 4. Surface Modifications of Metallic Biomaterials Using Composite Substances

**Figure 2:**
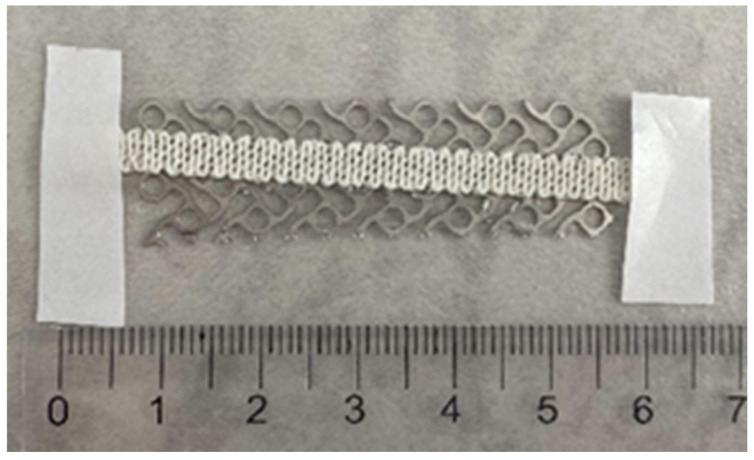
Hydroxyapatite and collagen structure 3D-printed on a titanium mesh. Date from Ref. [5].

Titanium mesh implants—metal structures with high physical resistance—are not ideal for osteogenic integration, as cell adhesion is suboptimal on materials with low surface roughness, poor wettability, and no porosity [89]. As illustrated in Figure 2, hydroxyapatite HA [90] and collagen [91] can be 3D-printed on titanium meshes to enhance surface roughness and osteointegration [87].

In conducted in vitro studies, these additive manufacturing implants resulted in improved in vitro mineralization [87]. However, no in vivo human studies have been conducted, and no definitive conclusions regarding their usage can be drawn.

## 5. Prospects

Orbital wall reconstruction remains a significant challenge due to the anatomical particularities of the orbit, high functional and aesthetic expectations, the unpredictability of lesion progression over the long term, and the potential need for iterative operations. The wide variety of biomaterials used over time—resorbable/non-resorbable; autologous, xenogeneic, or alloplastic; standard versus customized titanium mesh; and coated or uncoated—demonstrates that no ideal material exists. The new generation of biomaterials is expected to be more compatible and more bioactive, with increased capacities for bone regeneration [92]. Injectable hydrogels (IHs) reinforced with versatile nanomaterials (NMs) that have high-mechanical-performance properties could provide immunomodulatory bioactive characteristics, promote osteo- and angiogenesis, and exhibit antibacterial effects [93]. Geometric versatility—adaptive to any type of lesion—would render these innovative materials a perfect solution in the future [94]. The development of 3D nanostructured, biocompatible, on-site printable materials now appears to be an achievable goal [95]. However, further studies are needed to validate the clinical application of these emerging technologies and materials.

**Figure 3:**
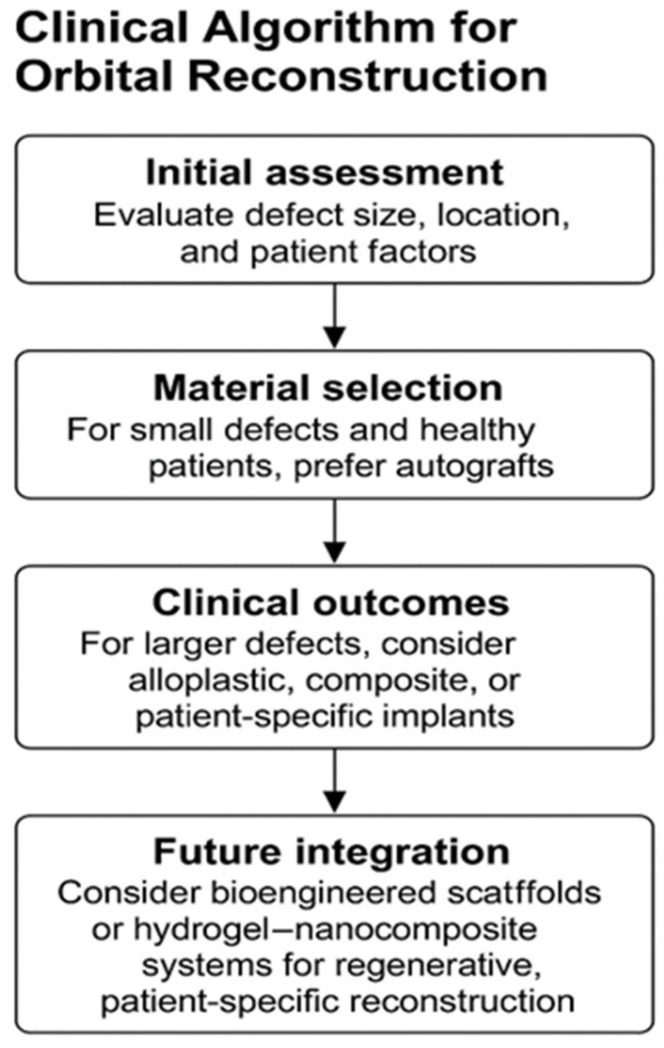
Clinical decision algorithm for selecting orbital wall reconstruction materials.

There are no universal or miraculous solutions for all patients; the decision to use a specific type of material for orbital wall reconstruction must take into account multiple factors: the size of the bone defect, the patient’s age and healing potential, the risk of infection (such as contaminated wounds or communication with sinuses), the availability of 3D-printing and facilities for producing customized implants. These are the reasons why the use of a logical algorithm for selecting the optimal reconstructive material is necessary (Figure 3).

The algorithm summarizes practical pathways for material selection in post-traumatic orbital wall reconstruction, integrating defect characteristics, patient factors, and material properties.

Despite rapid technological progress, several barriers hinder the transition of bioengineered and nanostructured materials from laboratory to clinical use. Regulatory approval requires robust, multi-center data on biocompatibility and long-term safety, while sterilization and storage standards for hybrid materials remain under development. Technical barriers include limited intraoperative customization in resource-limited hospitals. Additionally, high production costs, the need for specialized imaging and 3D printing facilities, and the steep learning curve for digital planning restrict routine adoption. Training programs and cost-reduction strategies will be crucial for broader clinical integration.

## 6. Conclusions

Despite major advances, post-traumatic orbital wall reconstruction still faces significant challenges. Current materials remain suboptimal, with limitations related to donor-site morbidity, resorption, infection risk, limited mechanical performance, and lack of long-term biocompatibility. No single solution fulfills all biomechanical and biological requirements for orbital wall repair.

Future directions are moving toward the integration of bioengineering, additive manufacturing, and regenerative medicine. Smart scaffolds, 3D-printed patient-specific implants, and bioactive coatings capable of modulating immune responses and promoting osteogenesis are expected to transform clinical practice. These technologies, coupled with cost reduction and real-time “point-of-care” printing, represent the most promising path toward achieving functional and aesthetic restoration in orbital reconstruction.

## Figures and Tables

**Table 1:** Comparative overview of biomaterials used in orbital wall reconstruction.

Material Category	Representative Examples	Main Advantages	Principal Limitations	Clinical Remarks
Autologous tissues	Calvarial bone, iliac crest, rib, nasal cartilage	Excellent biocompatibility; osteoconductive; low infection rate; readily available	Donor-site morbidity; limited quantity; unpredictable resorption	Best suited for small to medium defects; declining use with advent of alloplasts
Allografts/Xenografts	Lyophilized dura, demineralized bone matrix, porcine collagen	Avoids donor site surgery; biological integration possible	Risk of disease transmission; immune reaction; variable resorption	Use restricted; limited long-term data
Metals	Titanium mesh, titanium plates	High mechanical strength; stable volume; radiopaque; 3D-printing feasible	Rigid; possible cold conduction; expensive; may require removal	Widely used for large or complex defects; custom meshes available
Polymers (non-resorbable)	Porous polyethylene (Medpor), silicone, PTFE	Easily shaped; promotes fibrovascular ingrowth; good stability	Risk of late infection or extrusion; non-resorbable	Common for medium defects; careful asepsis required
Polymers (resorbable)	PLLA, PDS, PGA, copolymers	Eliminates second surgery; avoids growth restriction in children	Limited strength; unpredictable degradation; local inflammation possible	Preferred in pediatric or small orbital defects
Composites/Bioactive ceramics	HA-polymer composites, calcium phosphate ceramics	Osteoconductive; good stability; customizable; radiopaque	Brittle; expensive; limited clinical validation	Promising in hybrid 3D-printed and scaffold-based reconstructions

Note: The table summarizes key properties, advantages, and limitations of major classes of orbital reconstructive materials based on clinical and experimental data reported in the literature. Date from Refs. [5,6].

**Table 2:** Osteogenic effects of bioactive peptides in bone regeneration.

Bioactive Peptide	Function
PepGen P-15	Promoted: extracellular matrix production; proliferation and osteogenic differentiation; cell attachment, migration, and survival
Arginine-glycine-aspartic acid (RGD)	Promoted: proliferation, mineralization, and osteogenic differentiation; cell attachment and survival
Ser-Val—Val-Tyr-Gly-Leu-Arg (SVVYGLR)	Promoted: proliferation and neovascularization; angiogenesis and osteogenesis; adhesion; migration; tube formation of endothelial cells
Gly-Phel-Hydroxy-proline-arginine (GFGOER)	Promoted: differentiation, bone regeneration, osseointegration
Collagen binding motif (CBM)	Promoted: bone-related cell adhesion and growth; osteogenic differentiation
Fibronectin-derived peptides (FN-derived peptides)	Promoted: bone-related cell spreading; adhesion and mineralization
P17-BMP-2	Promoted: bone repair; osteoblast differentiation and bone regeneration
P20-BMP-2 and P 24- BMP-2	Promoted: osteogenesis and differentiation of MSCs into osteoblasts
BMP-7 derived BFP-1	Enhanced: Ca^2+^ content in cells, ALP activity, bone regeneration

Date from Ref. [80].

## Data Availability

No new data were created or analyzed in this study. Data sharing is not applicable to this article.
